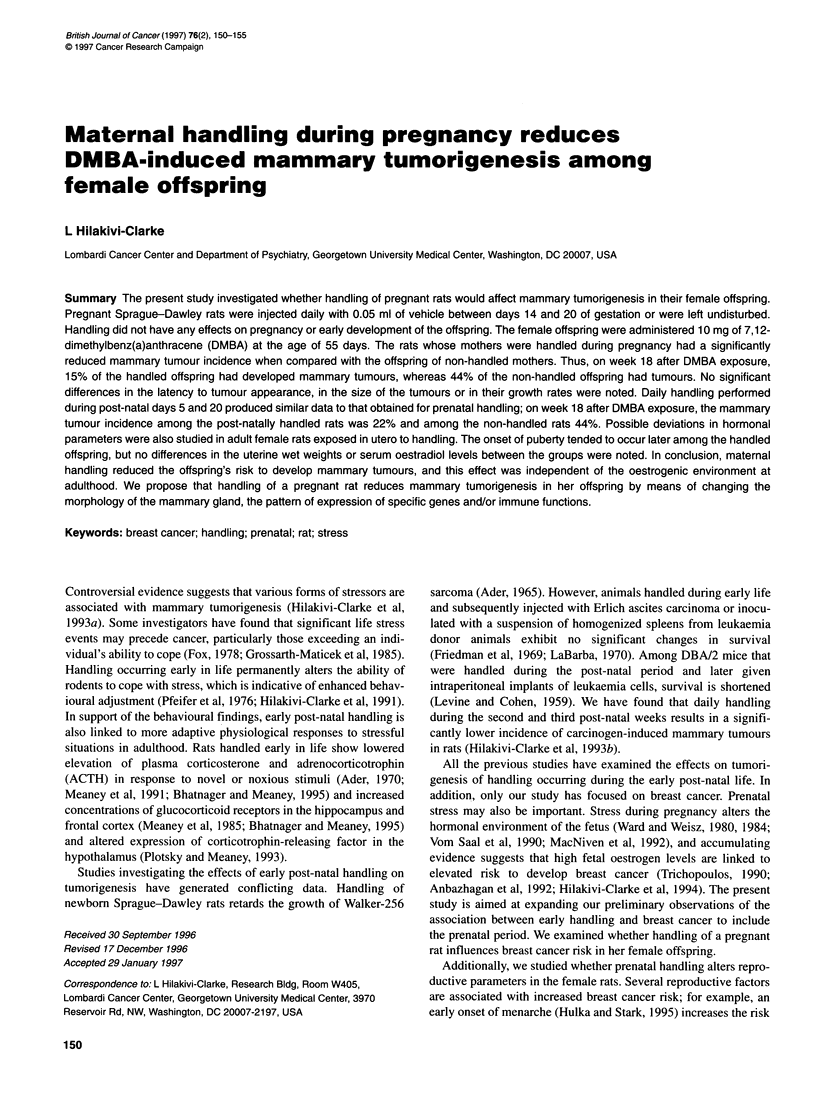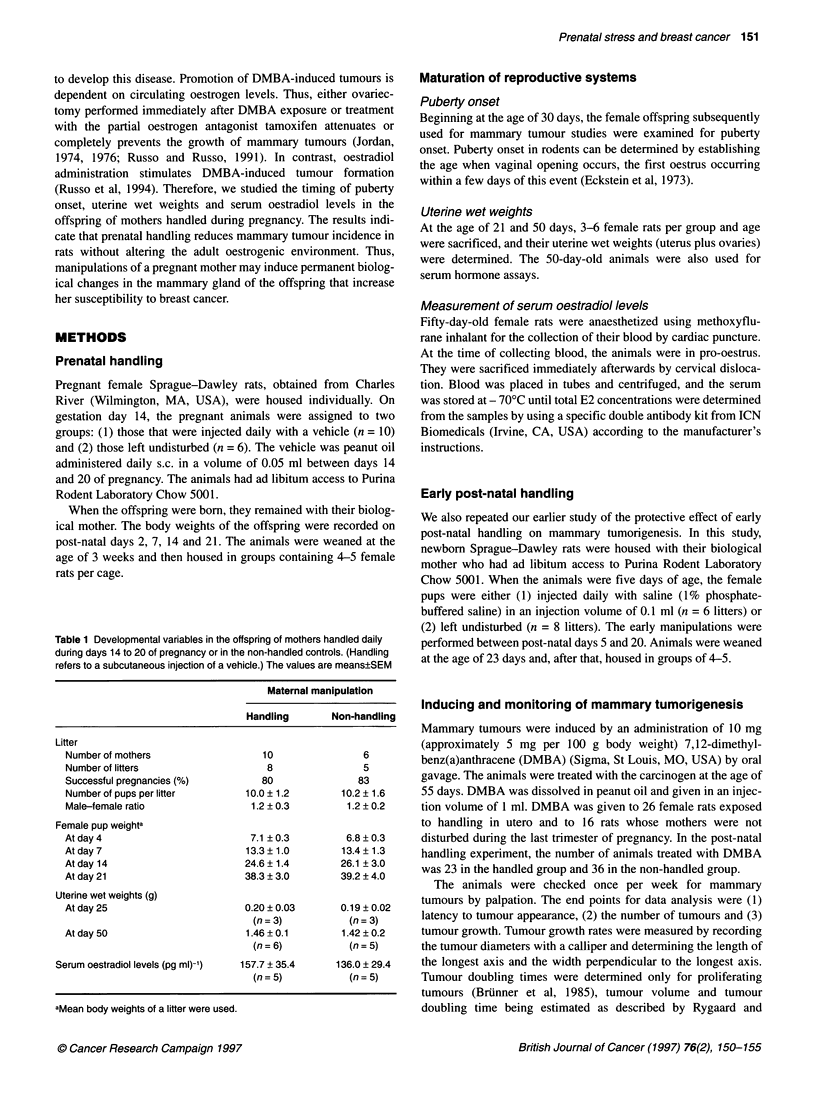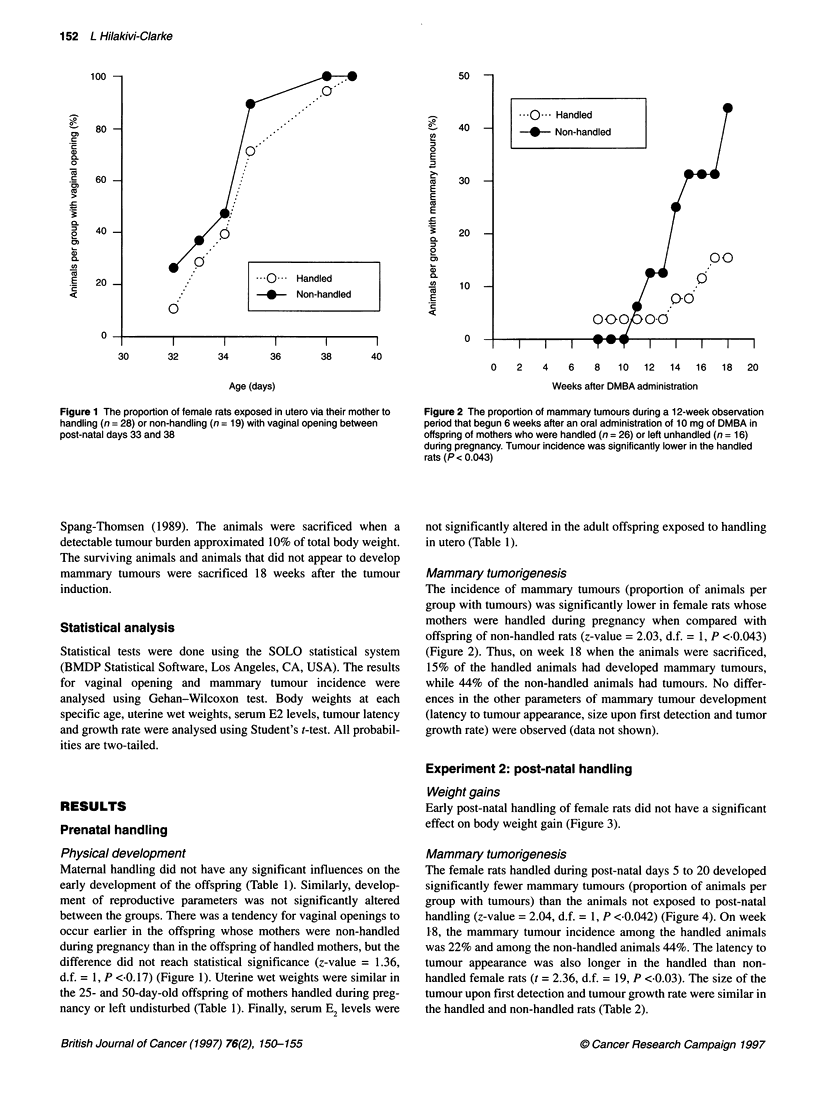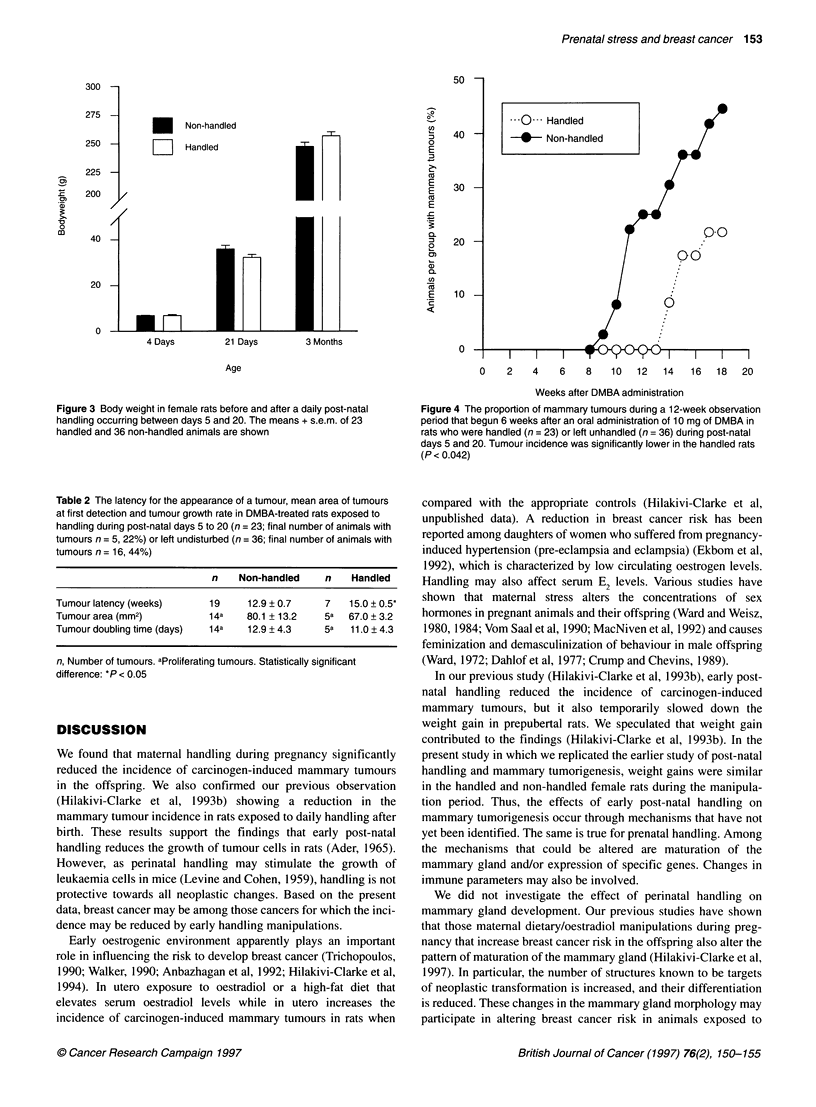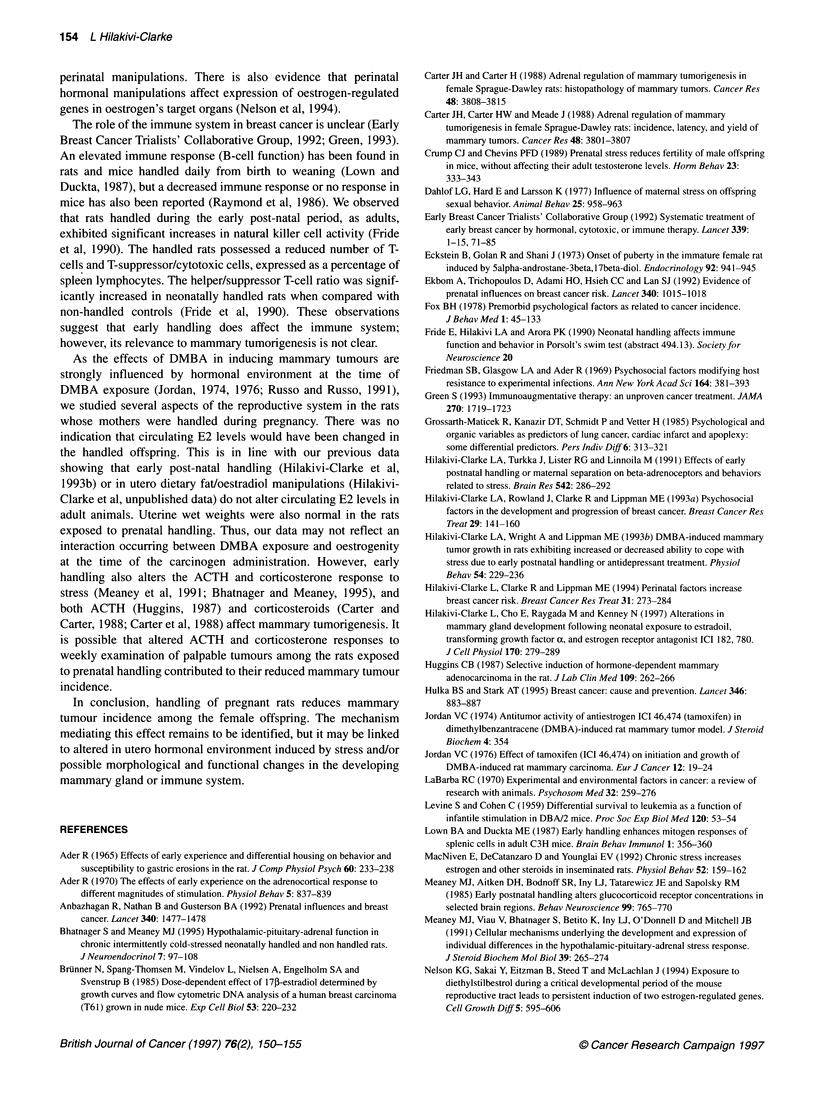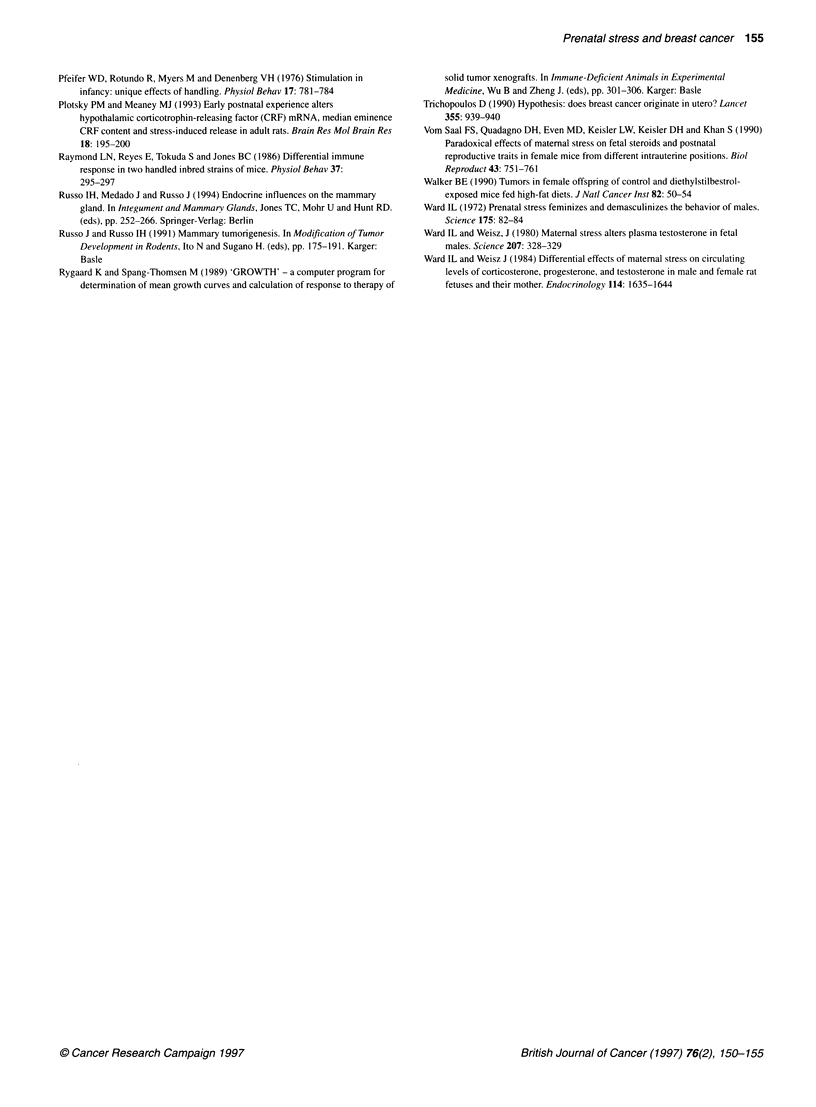# Maternal handling during pregnancy reduces DMBA-induced mammary tumorigenesis among female offspring.

**DOI:** 10.1038/bjc.1997.356

**Published:** 1997

**Authors:** L. Hilakivi-Clarke

**Affiliations:** Lombardi Cancer Center and Department of Psychiatry, Georgetown University Medical Center, Washington, DC 20007, USA.

## Abstract

The present study investigated whether handling of pregnant rats would affect mammary tumorigenesis in their female offspring. Pregnant Sprague-Dawley rats were injected daily with 0.05 ml of vehicle between days 14 and 20 of gestation or were left undisturbed. Handling did not have any effects on pregnancy or early development of the offspring. The female offspring were administered 10 mg of 7,12-dimethylbenz(a)anthracene (DMBA) at the age of 55 days. The rats whose mothers were handled during pregnancy had a significantly reduced mammary tumour incidence when compared with the offspring of non-handled mothers. Thus, on week 18 after DMBA exposure, 15% of the handled offspring had developed mammary tumours, whereas 44% of the non-handled offspring had tumours. No significant differences in the latency to tumour appearance, in the size of the tumours or in their growth rates were noted. Daily handling performed during post-natal days 5 and 20 produced similar data to that obtained for prenatal handling; on week 18 after DMBA exposure, the mammary tumour incidence among the post-natally handled rats was 22% and among the non-handled rats 44%. Possible deviations in hormonal parameters were also studied in adult female rats exposed in utero to handling. The onset of puberty tended to occur later among the handled offspring, but no differences in the uterine wet weights or serum oestradiol levels between the groups were noted. In conclusion, maternal handling reduced the offspring's risk to develop mammary tumours, and this effect was independent of the oestrogenic environment at adulthood. We propose that handling of a pregnant rat reduces mammary tumorigenesis in her offspring by means of changing the morphology of the mammary gland, the pattern of expression of specific genes and/or immune functions.


*British Journal of Cancer* (1997) **76**(2), 150–155
© 1997 Cancer Research Campaign

### Maternal handling during pregnancy reduces DMBA-induced mammary tumorigenesis among female offspring

**L Hilakivi-Clarke**

Lombardi Cancer Center and Department of Psychiatry, Georgetown University Medical Center, Washington, DC 20007, USA

**Summary** The present study investigated whether handling of pregnant rats would affect mammary tumorigenesis in their female offspring. Pregnant Sprague–Dawley rats were injected daily with 0.05 ml of vehicle between days 14 and 20 of gestation or were left undisturbed. Handling did not have any effects on pregnancy or early development of the offspring. The female offspring were administered 10 mg of 7,12-dimethylbenz(a)anthracene (DMBA) at the age of 55 days. The rats whose mothers were handled during pregnancy had a significantly reduced mammary tumour incidence when compared with the offspring of non-handled mothers. Thus, on week 18 after DMBA exposure, 15% of the handled offspring had developed mammary tumours, whereas 44% of the non-handled offspring had tumours. No significant differences in the latency to tumour appearance, in the size of the tumours or in their growth rates were noted. Daily handling performed during post-natal days 5 and 20 produced similar data to that obtained for prenatal handling; on week 18 after DMBA exposure, the mammary tumour incidence among the post-natally handled rats was 22% and among the non-handled rats 44%. Possible deviations in hormonal parameters were also studied in adult female rats exposed in utero to handling. The onset of puberty tended to occur later among the handled offspring, but no differences in the uterine wet weights or serum oestradiol levels between the groups were noted. In conclusion, maternal handling reduced the offspring's risk to develop mammary tumours, and this effect was independent of the oestrogenic environment at adulthood. We propose that handling of a pregnant rat reduces mammary tumorigenesis in her offspring by means of changing the morphology of the mammary gland, the pattern of expression of specific genes and/or immune functions.

**Keywords:** breast cancer; handling; prenatal; rat; stress

Controversial evidence suggests that various forms of stressors are associated with mammary tumorigenesis (Hilakivi-Clarke et al, 1993*a*). Some investigators have found that significant life stress events may precede cancer, particularly those exceeding an individual's ability to cope (Fox, 1978; Grossarth-Maticek et al, 1985). Handling occurring early in life permanently alters the ability of rodents to cope with stress, which is indicative of enhanced behavioural adjustment (Pfeifer et al, 1976; Hilakivi-Clarke et al, 1991). In support of the behavioural findings, early post-natal handling is also linked to more adaptive physiological responses to stressful situations in adulthood. Rats handled early in life show lowered elevation of plasma corticosterone and adrenocorticotrophin (ACTH) in response to novel or noxious stimuli (Ader, 1970; Meaney et al, 1991; Bhatnager and Meaney, 1995) and increased concentrations of glucocorticoid receptors in the hippocampus and frontal cortex (Meaney et al, 1985; Bhatnager and Meaney, 1995) and altered expression of corticotrophin-releasing factor in the hypothalamus (Plotsky and Meaney, 1993).

Studies investigating the effects of early post-natal handling on tumorigenesis have generated conflicting data. Handling of newborn Sprague–Dawley rats retards the growth of Walker-256 sarcoma (Ader, 1965). However, animals handled during early life and subsequently injected with Erlich ascites carcinoma or inoculated with a suspension of homogenized spleens from leukaemia donor animals exhibit no significant changes in survival (Friedman et al, 1969; LaBarba, 1970). Among DBA/2 mice that were handled during the post-natal period and later given intraperitoneal implants of leukaemia cells, survival is shortened (Levine and Cohen, 1959). We have found that daily handling during the second and third post-natal weeks results in a significantly lower incidence of carcinogen-induced mammary tumours in rats (Hilakivi-Clarke et al, 1993*b*).

All the previous studies have examined the effects on tumorigenesis of handling occurring during the early post-natal life. In addition, only our study has focused on breast cancer. Prenatal stress may also be important. Stress during pregnancy alters the hormonal environment of the fetus (Ward and Weisz, 1980, 1984; Vom Saal et al, 1990; MacNiven et al, 1992), and accumulating evidence suggests that high fetal oestrogen levels are linked to elevated risk to develop breast cancer (Trichopoulos, 1990; Anbazhagan et al, 1992; Hilakivi-Clarke et al, 1994). The present study is aimed at expanding our preliminary observations of the association between early handling and breast cancer to include the prenatal period. We examined whether handling of a pregnant rat influences breast cancer risk in her female offspring.

Additionally, we studied whether prenatal handling alters reproductive parameters in the female rats. Several reproductive factors are associated with increased breast cancer risk; for example, an early onset of menarche (Hulka and Stark, 1995) increases the risk

*Received 30 September 1996*
*Revised 17 December 1996*
*Accepted 29 January 1997*

*Correspondence to:* L Hilakivi-Clarke, Research Bldg, Room W405, Lombardi Cancer Center, Georgetown University Medical Center, 3970 Reservoir Rd, NW, Washington, DC 20007-2197, USA

to develop this disease. Promotion of DMBA-induced tumours is dependent on circulating oestrogen levels. Thus, either ovariectomy performed immediately after DMBA exposure or treatment with the partial oestrogen antagonist tamoxifen attenuates or completely prevents the growth of mammary tumours (Jordan, 1974, 1976; Russo and Russo, 1991). In contrast, oestradiol administration stimulates DMBA-induced tumour formation (Russo et al, 1994). Therefore, we studied the timing of puberty onset, uterine wet weights and serum oestradiol levels in the offspring of mothers handled during pregnancy. The results indicate that prenatal handling reduces mammary tumour incidence in rats without altering the adult oestrogenic environment. Thus, manipulations of a pregnant mother may induce permanent biological changes in the mammary gland of the offspring that increase her susceptibility to breast cancer.

### METHODS

### Prenatal handling

Pregnant female Sprague–Dawley rats, obtained from Charles River (Wilmington, MA, USA), were housed individually. On gestation day 14, the pregnant animals were assigned to two groups: (1) those that were injected daily with a vehicle ($n$ = 10) and (2) those left undisturbed ($n$ = 6). The vehicle was peanut oil administered daily s.c. in a volume of 0.05 ml between days 14 and 20 of pregnancy. The animals had ad libitum access to Purina Rodent Laboratory Chow 5001.

When the offspring were born, they remained with their biological mother. The body weights of the offspring were recorded on post-natal days 2, 7, 14 and 21. The animals were weaned at the age of 3 weeks and then housed in groups containing 4–5 female rats per cage.

**Table 1** Developmental variables in the offspring of mothers handled daily during days 14 to 20 of pregnancy or in the non-handled controls. (Handling refers to a subcutaneous injection of a vehicle.) The values are means±SEM

| | Maternal manipulation | |
|---|---|---|
| | **Handling** | **Non-handling** |
| Litter | | |
| Number of mothers | 10 | 6 |
| Number of litters | 8 | 5 |
| Successful pregnancies (%) | 80 | 83 |
| Number of pups per litter | 10.0 ± 1.2 | 10.2 ± 1.6 |
| Male–female ratio | 1.2 ± 0.3 | 1.2 ± 0.2 |
| Female pup weight[a] | | |
| At day 4 | 7.1 ± 0.3 | 6.8 ± 0.3 |
| At day 7 | 13.3 ± 1.0 | 13.4 ± 1.3 |
| At day 14 | 24.6 ± 1.4 | 26.1 ± 3.0 |
| At day 21 | 38.3 ± 3.0 | 39.2 ± 4.0 |
| Uterine wet weights (g) | | |
| At day 25 | 0.20 ± 0.03 ($n$ = 3) | 0.19 ± 0.02 ($n$ = 3) |
| At day 50 | 1.46 ± 0.1 ($n$ = 6) | 1.42 ± 0.2 ($n$ = 5) |
| Serum oestradiol levels (pg ml)$^{-1}$) | 157.7 ± 35.4 ($n$ = 5) | 136.0 ± 29.4 ($n$ = 5) |

[a]Mean body weights of a litter were used.

### Maturation of reproductive systems

### Puberty onset

Beginning at the age of 30 days, the female offspring subsequently used for mammary tumour studies were examined for puberty onset. Puberty onset in rodents can be determined by establishing the age when vaginal opening occurs, the first oestrus occurring within a few days of this event (Eckstein et al, 1973).

### Uterine wet weights

At the age of 21 and 50 days, 3–6 female rats per group and age were sacrificed, and their uterine wet weights (uterus plus ovaries) were determined. The 50-day-old animals were also used for serum hormone assays.

### Measurement of serum oestradiol levels

Fifty-day-old female rats were anaesthetized using methoxyflurane inhalant for the collection of their blood by cardiac puncture. At the time of collecting blood, the animals were in pro-oestrus. They were sacrificed immediately afterwards by cervical dislocation. Blood was placed in tubes and centrifuged, and the serum was stored at – 70°C until total E2 concentrations were determined from the samples by using a specific double antibody kit from ICN Biomedicals (Irvine, CA, USA) according to the manufacturer's instructions.

### Early post-natal handling

We also repeated our earlier study of the protective effect of early post-natal handling on mammary tumorigenesis. In this study, newborn Sprague–Dawley rats were housed with their biological mother who had ad libitum access to Purina Rodent Laboratory Chow 5001. When the animals were five days of age, the female pups were either (1) injected daily with saline (1% phosphate-buffered saline) in an injection volume of 0.1 ml ($n$ = 6 litters) or (2) left undisturbed ($n$ = 8 litters). The early manipulations were performed between post-natal days 5 and 20. Animals were weaned at the age of 23 days and, after that, housed in groups of 4–5.

### Inducing and monitoring of mammary tumorigenesis

Mammary tumours were induced by an administration of 10 mg (approximately 5 mg per 100 g body weight) 7,12-dimethylbenz(a)anthracene (DMBA) (Sigma, St Louis, MO, USA) by oral gavage. The animals were treated with the carcinogen at the age of 55 days. DMBA was dissolved in peanut oil and given in an injection volume of 1 ml. DMBA was given to 26 female rats exposed to handling in utero and to 16 rats whose mothers were not disturbed during the last trimester of pregnancy. In the post-natal handling experiment, the number of animals treated with DMBA was 23 in the handled group and 36 in the non-handled group.

The animals were checked once per week for mammary tumours by palpation. The end points for data analysis were (1) latency to tumour appearance, (2) the number of tumours and (3) tumour growth. Tumour growth rates were measured by recording the tumour diameters with a calliper and determining the length of the longest axis and the width perpendicular to the longest axis. Tumour doubling times were determined only for proliferating tumours (Brünner et al, 1985), tumour volume and tumour doubling time being estimated as described by Rygaard and

© Cancer Research Campaign 1997

**Figure 1** The proportion of female rats exposed in utero via their mother to handling ($n = 28$) or non-handling ($n = 19$) with vaginal opening between post-natal days 33 and 38

**Figure 2** The proportion of mammary tumours during a 12-week observation period that begun 6 weeks after an oral administration of 10 mg of DMBA in offspring of mothers who were handled ($n = 26$) or left unhandled ($n = 16$) during pregnancy. Tumour incidence was significantly lower in the handled rats ($P < 0.043$)

Spang-Thomsen (1989). The animals were sacrificed when a detectable tumour burden approximated 10% of total body weight. The surviving animals and animals that did not appear to develop mammary tumours were sacrificed 18 weeks after the tumour induction.

### Statistical analysis

Statistical tests were done using the SOLO statistical system (BMDP Statistical Software, Los Angeles, CA, USA). The results for vaginal opening and mammary tumour incidence were analysed using Gehan–Wilcoxon test. Body weights at each specific age, uterine wet weights, serum E2 levels, tumour latency and growth rate were analysed using Student's $t$-test. All probabilities are two-tailed.

### RESULTS

### Prenatal handling

### *Physical development*

Maternal handling did not have any significant influences on the early development of the offspring (Table 1). Similarly, development of reproductive parameters was not significantly altered between the groups. There was a tendency for vaginal openings to occur earlier in the offspring whose mothers were non-handled during pregnancy than in the offspring of handled mothers, but the difference did not reach statistical significance ($z$-value = 1.36, d.f. = 1, $P <\cdot 0.17$) (Figure 1). Uterine wet weights were similar in the 25- and 50-day-old offspring of mothers handled during pregnancy or left undisturbed (Table 1). Finally, serum $E_2$ levels were not significantly altered in the adult offspring exposed to handling in utero (Table 1).

### *Mammary tumorigenesis*

The incidence of mammary tumours (proportion of animals per group with tumours) was significantly lower in female rats whose mothers were handled during pregnancy when compared with offspring of non-handled rats ($z$-value = 2.03, d.f. = 1, $P <\cdot 0.043$) (Figure 2). Thus, on week 18 when the animals were sacrificed, 15% of the handled animals had developed mammary tumours, while 44% of the non-handled animals had tumours. No differences in the other parameters of mammary tumour development (latency to tumour appearance, size upon first detection and tumor growth rate) were observed (data not shown).

### Experiment 2: post-natal handling

### *Weight gains*

Early post-natal handling of female rats did not have a significant effect on body weight gain (Figure 3).

### *Mammary tumorigenesis*

The female rats handled during post-natal days 5 to 20 developed significantly fewer mammary tumours (proportion of animals per group with tumours) than the animals not exposed to post-natal handling ($z$-value = 2.04, d.f. = 1, $P <\cdot 0.042$) (Figure 4). On week 18, the mammary tumour incidence among the handled animals was 22% and among the non-handled animals 44%. The latency to tumour appearance was also longer in the handled than non-handled female rats ($t$ = 2.36, d.f. = 19, $P <\cdot 0.03$). The size of the tumour upon first detection and tumour growth rate were similar in the handled and non-handled rats (Table 2).

© Cancer Research Campaign 1997

**Figure 3** Body weight in female rats before and after a daily post-natal handling occurring between days 5 and 20. The means + s.e.m. of 23 handled and 36 non-handled animals are shown

**Figure 4** The proportion of mammary tumours during a 12-week observation period that begun 6 weeks after an oral administration of 10 mg of DMBA in rats who were handled ($n$ = 23) or left unhandled ($n$ = 36) during post-natal days 5 and 20. Tumour incidence was significantly lower in the handled rats ($P$ < 0.042)

**Table 2** The latency for the appearance of a tumour, mean area of tumours at first detection and tumour growth rate in DMBA-treated rats exposed to handling during post-natal days 5 to 20 ($n$ = 23; final number of animals with tumours $n$ = 5, 22%) or left undisturbed ($n$ = 36; final number of animals with tumours $n$ = 16, 44%)

| | $n$ | Non-handled | $n$ | Handled |
|---|---|---|---|---|
| Tumour latency (weeks) | 19 | 12.9 ± 0.7 | 7 | 15.0 ± 0.5* |
| Tumour area (mm$^2$) | 14[a] | 80.1 ± 13.2 | 5[a] | 67.0 ± 3.2 |
| Tumour doubling time (days) | 14[a] | 12.9 ± 4.3 | 5[a] | 11.0 ± 4.3 |

$n$, Number of tumours. [a]Proliferating tumours. Statistically significant difference: *$P$ < 0.05

### DISCUSSION

We found that maternal handling during pregnancy significantly reduced the incidence of carcinogen-induced mammary tumours in the offspring. We also confirmed our previous observation (Hilakivi-Clarke et al, 1993b) showing a reduction in the mammary tumour incidence in rats exposed to daily handling after birth. These results support the findings that early post-natal handling reduces the growth of tumour cells in rats (Ader, 1965). However, as perinatal handling may stimulate the growth of leukaemia cells in mice (Levine and Cohen, 1959), handling is not protective towards all neoplastic changes. Based on the present data, breast cancer may be among those cancers for which the incidence may be reduced by early handling manipulations.

Early oestrogenic environment apparently plays an important role in influencing the risk to develop breast cancer (Trichopoulos, 1990; Walker, 1990; Anbazhagan et al, 1992; Hilakivi-Clarke et al, 1994). In utero exposure to oestradiol or a high-fat diet that elevates serum oestradiol levels while in utero increases the incidence of carcinogen-induced mammary tumours in rats when compared with the appropriate controls (Hilakivi-Clarke et al, unpublished data). A reduction in breast cancer risk has been reported among daughters of women who suffered from pregnancy-induced hypertension (pre-eclampsia and eclampsia) (Ekbom et al, 1992), which is characterized by low circulating oestrogen levels. Handling may also affect serum $E_2$ levels. Various studies have shown that maternal stress alters the concentrations of sex hormones in pregnant animals and their offspring (Ward and Weisz, 1980, 1984; Vom Saal et al, 1990; MacNiven et al, 1992) and causes feminization and demasculinization of behaviour in male offspring (Ward, 1972; Dahlof et al, 1977; Crump and Chevins, 1989).

In our previous study (Hilakivi-Clarke et al, 1993b), early post-natal handling reduced the incidence of carcinogen-induced mammary tumours, but it also temporarily slowed down the weight gain in prepubertal rats. We speculated that weight gain contributed to the findings (Hilakivi-Clarke et al, 1993b). In the present study in which we replicated the earlier study of post-natal handling and mammary tumorigenesis, weight gains were similar in the handled and non-handled female rats during the manipulation period. Thus, the effects of early post-natal handling on mammary tumorigenesis occur through mechanisms that have not yet been identified. The same is true for prenatal handling. Among the mechanisms that could be altered are maturation of the mammary gland and/or expression of specific genes. Changes in immune parameters may also be involved.

We did not investigate the effect of perinatal handling on mammary gland development. Our previous studies have shown that those maternal dietary/oestradiol manipulations during pregnancy that increase breast cancer risk in the offspring also alter the pattern of maturation of the mammary gland (Hilakivi-Clarke et al, 1997). In particular, the number of structures known to be targets of neoplastic transformation is increased, and their differentiation is reduced. These changes in the mammary gland morphology may participate in altering breast cancer risk in animals exposed to perinatal manipulations. There is also evidence that perinatal hormonal manipulations affect expression of oestrogen-regulated genes in oestrogen's target organs (Nelson et al, 1994).

The role of the immune system in breast cancer is unclear (Early Breast Cancer Trialists' Collaborative Group, 1992; Green, 1993). An elevated immune response (B-cell function) has been found in rats and mice handled daily from birth to weaning (Lown and Duckta, 1987), but a decreased immune response or no response in mice has also been reported (Raymond et al, 1986). We observed that rats handled during the early post-natal period, as adults, exhibited significant increases in natural killer cell activity (Fride et al, 1990). The handled rats possessed a reduced number of T-cells and T-suppressor/cytotoxic cells, expressed as a percentage of spleen lymphocytes. The helper/suppressor T-cell ratio was significantly increased in neonatally handled rats when compared with non-handled controls (Fride et al, 1990). These observations suggest that early handling does affect the immune system; however, its relevance to mammary tumorigenesis is not clear.

As the effects of DMBA in inducing mammary tumours are strongly influenced by hormonal environment at the time of DMBA exposure (Jordan, 1974, 1976; Russo and Russo, 1991), we studied several aspects of the reproductive system in the rats whose mothers were handled during pregnancy. There was no indication that circulating E2 levels would have been changed in the handled offspring. This is in line with our previous data showing that early post-natal handling (Hilakivi-Clarke et al, 1993b) or in utero dietary fat/oestradiol manipulations (Hilakivi-Clarke et al, unpublished data) do not alter circulating E2 levels in adult animals. Uterine wet weights were also normal in the rats exposed to prenatal handling. Thus, our data may not reflect an interaction occurring between DMBA exposure and oestrogenity at the time of the carcinogen administration. However, early handling also alters the ACTH and corticosterone response to stress (Meaney et al, 1991; Bhatnager and Meaney, 1995), and both ACTH (Huggins, 1987) and corticosteroids (Carter and Carter, 1988; Carter et al, 1988) affect mammary tumorigenesis. It is possible that altered ACTH and corticosterone responses to weekly examination of palpable tumours among the rats exposed to prenatal handling contributed to their reduced mammary tumour incidence.

In conclusion, handling of pregnant rats reduces mammary tumour incidence among the female offspring. The mechanism mediating this effect remains to be identified, but it may be linked to altered in utero hormonal environment induced by stress and/or possible morphological and functional changes in the developing mammary gland or immune system.

### REFERENCES

Ader R (1965) Effects of early experience and differential housing on behavior and susceptibility to gastric erosions in the rat. *J Comp Physiol Psych* **60**: 233–238

Ader R (1970) The effects of early experience on the adrenocortical response to different magnitudes of stimulation. *Physiol Behav* **5**: 837–839

Anbazhagan R, Nathan B and Gusterson BA (1992) Prenatal influences and breast cancer. *Lancet* **340**: 1477–1478

Bhatnager S and Meaney MJ (1995) Hypothalamic-pituitary-adrenal function in chronic intermittently cold-stressed neonatally handled and non handled rats. *J Neuroendocrinol* **7**: 97–108

Brünner N, Spang-Thomsen M, Vindelov L, Nielsen A, Engelholm SA and Svenstrup B (1985) Dose-dependent effect of 17β-estradiol determined by growth curves and flow cytometric DNA analysis of a human breast carcinoma (T61) grown in nude mice. *Exp Cell Biol* **53**: 220–232

Carter JH and Carter H (1988) Adrenal regulation of mammary tumorigenesis in female Sprague-Dawley rats: histopathology of mammary tumors. *Cancer Res* **48**: 3808–3815

Carter JH, Carter HW and Meade J (1988) Adrenal regulation of mammary tumorigenesis in female Sprague-Dawley rats: incidence, latency, and yield of mammary tumors. *Cancer Res* **48**: 3801–3807

Crump CJ and Chevins PFD (1989) Prenatal stress reduces fertility of male offspring in mice, without affecting their adult testosterone levels. *Horm Behav* **23**: 333–343

Dahlof LG, Hard E and Larsson K (1977) Influence of maternal stress on offspring sexual behavior. *Animal Behav* **25**: 958–963

Early Breast Cancer Trialists' Collaborative Group (1992) Systematic treatment of early breast cancer by hormonal, cytotoxic, or immune therapy. *Lancet* **339**: 1–15, 71–85

Eckstein B, Golan R and Shani J (1973) Onset of puberty in the immature female rat induced by 5alpha-androstane-3beta,17beta-diol. *Endocrinology* **92**: 941–945

Ekbom A, Trichopoulos D, Adami HO, Hsieh CC and Lan SJ (1992) Evidence of prenatal influences on breast cancer risk. *Lancet* **340**: 1015–1018

Fox BH (1978) Premorbid psychological factors as related to cancer incidence. *J Behav Med* **1**: 45–133

Fride E, Hilakivi LA and Arora PK (1990) Neonatal handling affects immune function and behavior in Porsolt's swim test (abstract 494.13). *Society for Neuroscience* **20**

Friedman SB, Glasgow LA and Ader R (1969) Psychosocial factors modifying host resistance to experimental infections. *Ann New York Acad Sci* **164**: 381–393

Green S (1993) Immunoaugmentative therapy: an unproven cancer treatment. *JAMA* **270**: 1719–1723

Grossarth-Maticek R, Kanazir DT, Schmidt P and Vetter H (1985) Psychological and organic variables as predictors of lung cancer, cardiac infarct and apoplexy: some differential predictors. *Pers Indiv Diff* **6**: 313–321

Hilakivi-Clarke LA, Turkka J, Lister RG and Linnoila M (1991) Effects of early postnatal handling or maternal separation on beta-adrenoceptors and behaviors related to stress. *Brain Res* **542**: 286–292

Hilakivi-Clarke LA, Rowland J, Clarke R and Lippman ME (1993*a*) Psychosocial factors in the development and progression of breast cancer. *Breast Cancer Res Treat* **29**: 141–160

Hilakivi-Clarke LA, Wright A and Lippman ME (1993*b*) DMBA-induced mammary tumor growth in rats exhibiting increased or decreased ability to cope with stress due to early postnatal handling or antidepressant treatment. *Physiol Behav* **54**: 229–236

Hilakivi-Clarke L, Clarke R and Lippman ME (1994) Perinatal factors increase breast cancer risk. *Breast Cancer Res Treat* **31**: 273–284

Hilakivi-Clarke L, Cho E, Raygada M and Kenney N (1997) Alterations in mammary gland development following neonatal exposure to estradoil, transforming growth factor α, and estrogen receptor antagonist ICI 182, 780. *J Cell Physiol* **170**: 279–289

Huggins CB (1987) Selective induction of hormone-dependent mammary adenocarcinoma in the rat. *J Lab Clin Med* **109**: 262–266

Hulka BS and Stark AT (1995) Breast cancer: cause and prevention. *Lancet* **346**: 883–887

Jordan VC (1974) Antitumor activity of antiestrogen ICI 46,474 (tamoxifen) in dimethylbenzantracene (DMBA)-induced rat mammary tumor model. *J Steroid Biochem* **4**: 354

Jordan VC (1976) Effect of tamoxifen (ICI 46,474) on initiation and growth of DMBA-induced rat mammary carcinoma. *Eur J Cancer* **12**: 19–24

LaBarba RC (1970) Experimental and environmental factors in cancer: a review of research with animals. *Psychosom Med* **32**: 259–276

Levine S and Cohen C (1959) Differential survival to leukemia as a function of infantile stimulation in DBA/2 mice. *Proc Soc Exp Biol Med* **120**: 53–54

Lown BA and Duckta ME (1987) Early handling enhances mitogen responses of splenic cells in adult C3H mice. *Brain Behav Immunol* **1**: 356–360

MacNiven E, DeCatanzaro D and Younglai EV (1992) Chronic stress increases estrogen and other steroids in inseminated rats. *Physiol Behav* **52**: 159–162

Meaney MJ, Aitken DH, Bodnoff SR, Iny LJ, Tatarewicz JE and Sapolsky RM (1985) Early postnatal handling alters glucocorticoid receptor concentrations in selected brain regions. *Behav Neuroscience* **99**: 765–770

Meaney MJ, Viau V, Bhatnager S, Betito K, Iny LJ, O'Donnell D and Mitchell JB (1991) Cellular mechanisms underlying the development and expression of individual differences in the hypothalamic-pituitary-adrenal stress response. *J Steroid Biochem Mol Biol* **39**: 265–274

Nelson KG, Sakai Y, Eitzman B, Steed T and McLachlan J (1994) Exposure to diethylstilbestrol during a critical developmental period of the mouse reproductive tract leads to persistent induction of two estrogen-regulated genes. *Cell Growth Diff* **5**: 595–606

© Cancer Research Campaign 1997

Pfeifer WD, Rotundo R, Myers M and Denenberg VH (1976) Stimulation in infancy: unique effects of handling. *Physiol Behav* **17**: 781–784

Plotsky PM and Meaney MJ (1993) Early postnatal experience alters hypothalamic corticotrophin-releasing factor (CRF) mRNA, median eminence CRF content and stress-induced release in adult rats. *Brain Res Mol Brain Res* **18**: 195–200

Raymond LN, Reyes E, Tokuda S and Jones BC (1986) Differential immune response in two handled inbred strains of mice. *Physiol Behav* **37**: 295–297

Russo IH, Medado J and Russo J (1994) Endocrine influences on the mammary gland. In *Integument and Mammary Glands*, Jones TC, Mohr U and Hunt RD. (eds), pp. 252–266. Springer-Verlag: Berlin

Russo J and Russo IH (1991) Mammary tumorigenesis. In *Modification of Tumor Development in Rodents*, Ito N and Sugano H. (eds), pp. 175–191. Karger: Basle

Rygaard K and Spang-Thomsen M (1989) 'GROWTH' – a computer program for determination of mean growth curves and calculation of response to therapy of solid tumor xenografts. In *Immune-Deficient Animals in Experimental Medicine*, Wu B and Zheng J. (eds), pp. 301–306. Karger: Basle

Trichopoulos D (1990) Hypothesis: does breast cancer originate in utero? *Lancet* **355**: 939–940

Vom Saal FS, Quadagno DH, Even MD, Keisler LW, Keisler DH and Khan S (1990) Paradoxical effects of maternal stress on fetal steroids and postnatal reproductive traits in female mice from different intrauterine positions. *Biol Reproduct* **43**: 751–761

Walker BE (1990) Tumors in female offspring of control and diethylstilbestrol-exposed mice fed high-fat diets. *J Natl Cancer Inst* **82**: 50–54

Ward IL (1972) Prenatal stress feminizes and demasculinizes the behavior of males. *Science* **175**: 82–84

Ward IL and Weisz, J (1980) Maternal stress alters plasma testosterone in fetal males. *Science* **207**: 328–329

Ward IL and Weisz J (1984) Differential effects of maternal stress on circulating levels of corticosterone, progesterone, and testosterone in male and female rat fetuses and their mother. *Endocrinology* **114**: 1635–1644

© Cancer Research Campaign 1997